# Supplementary figures and images for: Pulmonary inflammation and viral replication define distinct clinical outcomes in fatal cases of COVID-19
Source: PLoS Pathog. 2024 Jun 5;20(6):e1012222. doi: 10.1371/journal.ppat.1012222 (PMC11182505; doi:10.1371/journal.ppat.1012222)

Supplementary Fig. 1. Sá et al.

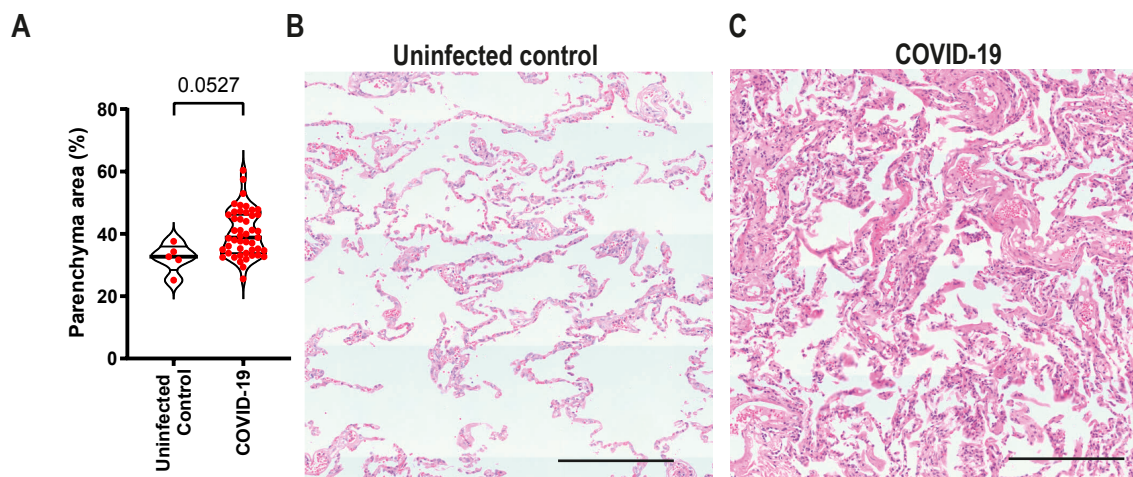

Supplement: S1 Fig — (A) The proportion of lung parenchyma area (loss of airspace) of COVID-19 patients and uninfected controls (benign area of the lungs from adenocarcinoma patients). (B-C) Representative images of H&E stain showing the lung parenchyma. Scale bars 200 μm. Each dot in the figure represents the value obtained from each individual. P-values are shown in the figures comparing the indicated groups, as determined by Mann–Whitney test. Data are represented as violin plots with median and quartiles. (PDF) [file ppat.1012222.s001.pdf]

Supplementary Fig. 2. Sá et al.

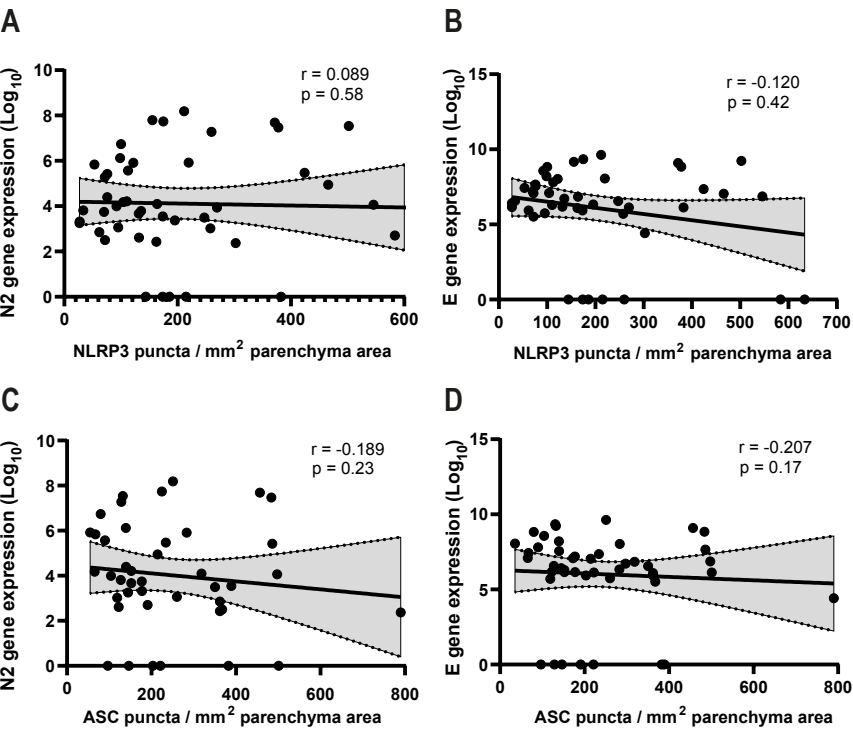

Supplement: S2 Fig — Spearman correlation of pulmonary viral load and inflammasome activation in 47 fatal COVID-19 patients. (A) Correlation of viral N2 with NLRP3 puncta per parenchyma area; (B) Correlation of viral E with NLRP3 puncta per parenchyma area; (C) Correlation of viral N2 with ASC puncta per parenchyma area; (D) Correlation of viral E with ASC puncta per parenchyma area. r and p-value are indicated in the figure. (PDF) [file ppat.1012222.s002.pdf]

Supplementary Fig. 3. Sá et al.

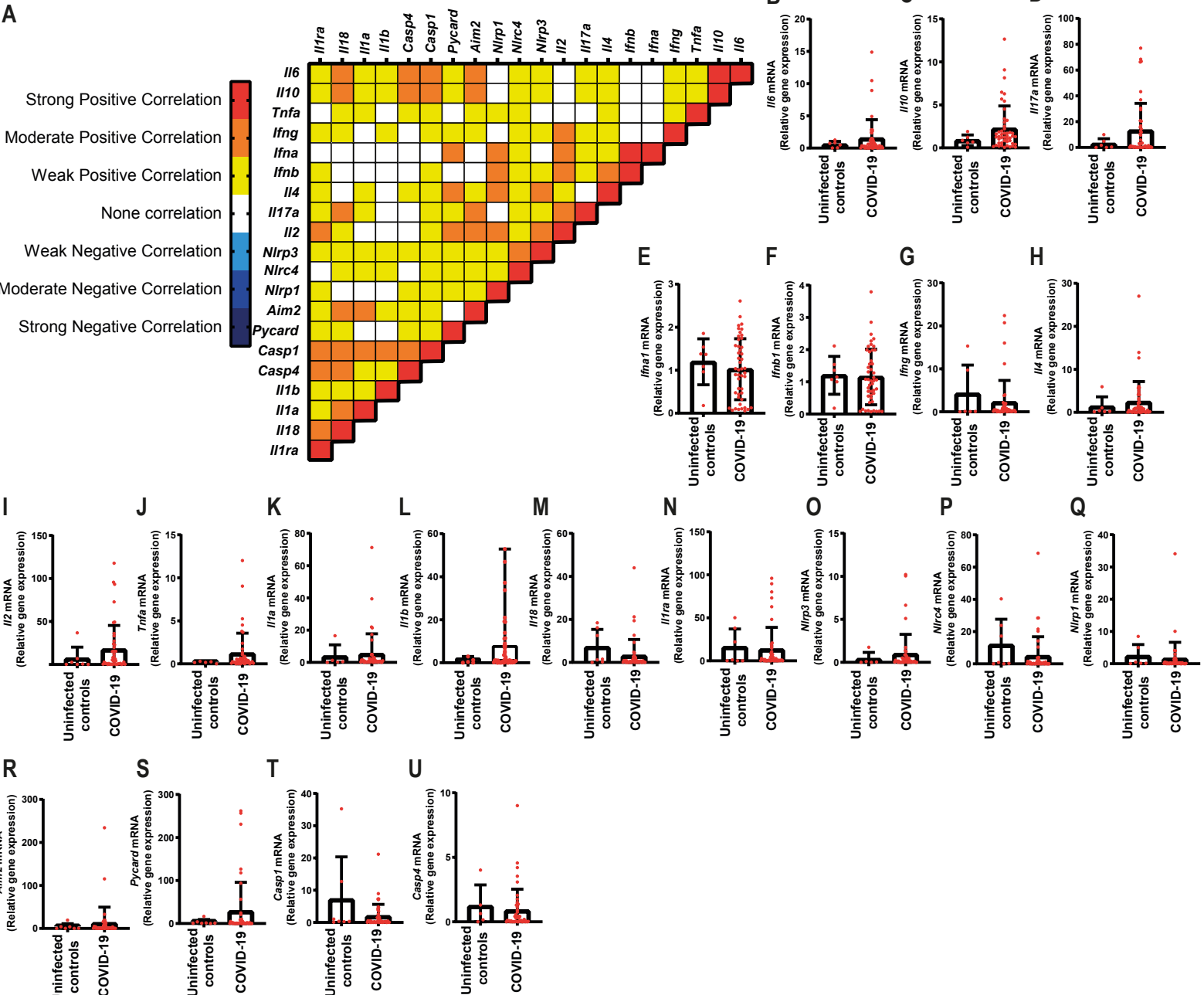

Supplement: S3 Fig — Correlation matrix of inflammasome and inflammatory gene expression in lung autopsy of 47 COVID-19 patients (A). Colors indicate correlation scores, categorized as positive strong correlation (r ≥ 0.70; red); moderate positive correlation (0.50 ≥ r ≤ 0.70; orange); weak positive correlation (0.30 ≥ r ≤ 0.50; yellow); negative strong correlation (r ≥ -0.70; dark blue); negative moderate correlation (-0.50 ≥ r ≤ -0.70; blue) or negative weak correlation (-0.30 ≥ r ≤ -0.50; light blue). Only correlations with p<0.05 are represented in the correlation matrix. (B-U) Expression of mRNA in the lung autopsies of COVID-19 patients and uninfected controls (benign area of the lungs from adenocarcinoma patients). Selected genes were Il6 (B), Il10 (C), Il17 (D), Ifna1 (E), Ifnb1 (F), Ifng (G), Il4 (H), Il2 (I), Tnfa (J), Il1a (K), Il1b (L), Il18 (M), I1ra (N), Nlrp3 (O), Nlrc4 (P), Nlrp1 (Q), Aim2 (R), Pycard (S), Casp1 (T), Casp4 (U). (PDF) [file ppat.1012222.s003.pdf]

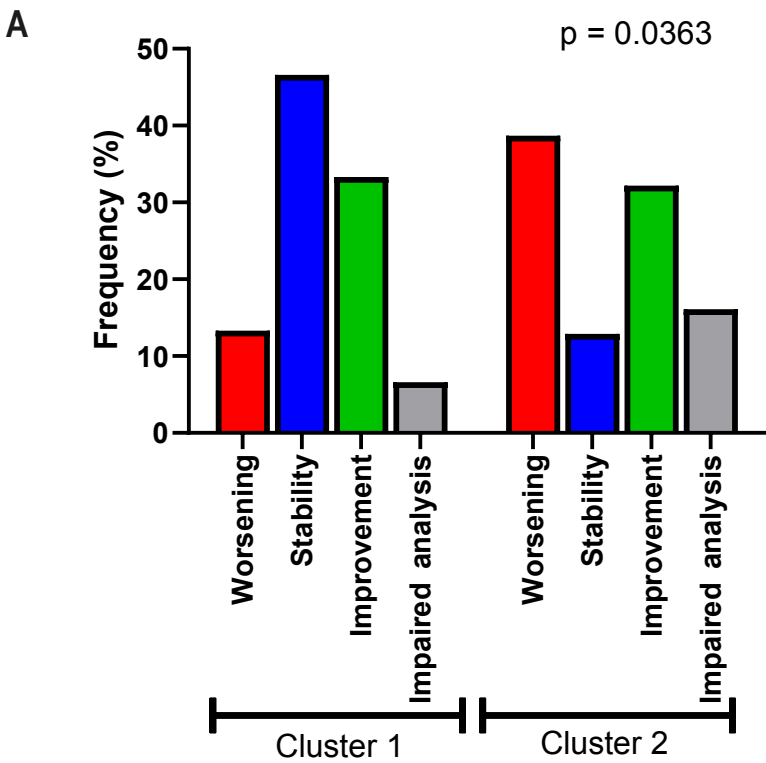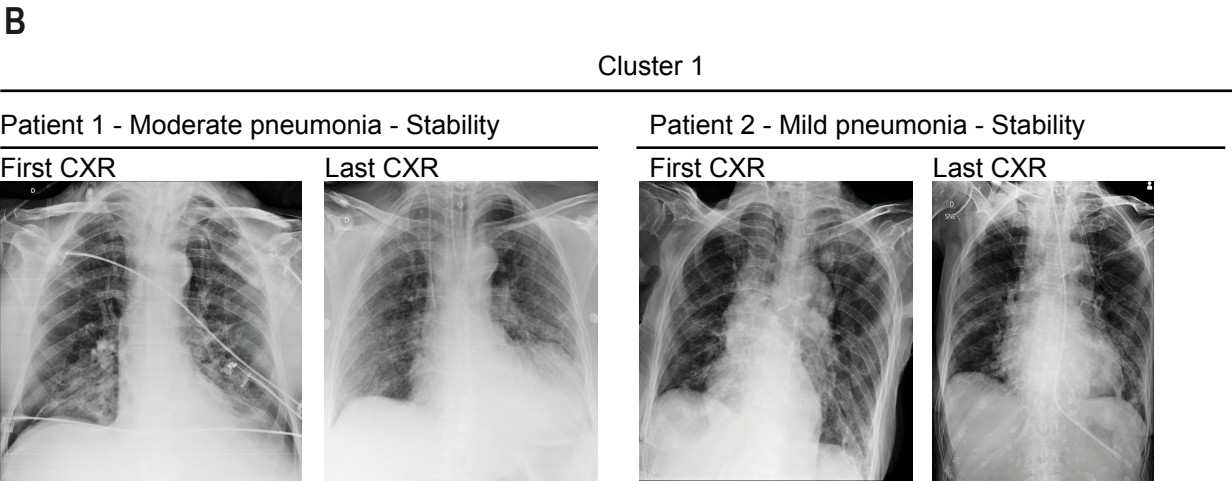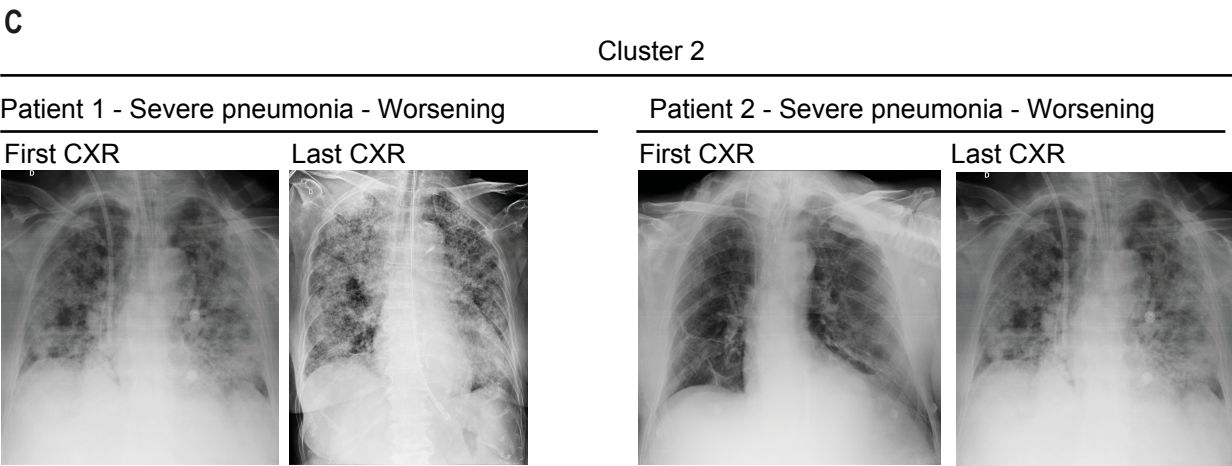

Supplement: S4 Fig — Analysis of the first and last Chest x-radiography (CXR) of 47 COVID-19 patients belonging to Cluster 1 (n = 15) and Cluster 2 (n = 31). (A) patients with reduced opacities (green), stability (blue), and increased opacities (red) comparing the fists and first CXR. Impaired analyses are shown in gray. Representative images of first and last CXR from two patients from Cluster 1, indicating stability in moderate and mild pneumonia (B) and two patients from Cluster 2, indicating worsening conditions in cases of severe pneumonia (C). (PDF) [file ppat.1012222.s004.pdf]
