# Supplementary material for: Pulmonary inflammation and viral replication define distinct clinical outcomes in fatal cases of COVID-19
Source: PLoS Pathog. 2024 Jun 5;20(6):e1012222. doi: 10.1371/journal.ppat.1012222 (PMC11182505; doi:10.1371/journal.ppat.1012222)
Supplement: S1 Table — (PDF) [file ppat.1012222.s005.pdf]

Supplementary Table 1. The list of primer sequences for real time-PCR

Gene	Sequence of primer (5'- 3')
Human *Nlrp3*	F: GGACTGAAGCACCTGTTGTGCA
	R: TCCTGAGTCTCCCAAGGCATTC
Human *Il1a*	F: TGTATGTGACTGCCCAAGATGAAG
	R: AGAGGAGGTTGGTCTCACTACC
Human *Il1ra*	F: ATGGAGGGAAGATGTGCCTGTC
	R: GTCCTGCTTTCTGTTCTCGCTC
Human *Nlrc4*	F: AGGTCCCACAACTCGTCAAGCT
	R: TGCTCACACGATTTCCCGCCAA
Human *Pycard*	F: AGCTCACCGCTAACGTGCTGC
	R: GCTTGGCTGCCGACTGAGGAG
Human *Nlrp1*	F: ATTGAGGGCAGGCAGCACAGAT
	R: CTCCTTCAGGTTTCTGGTGACC
Human *Casp1*	F: GCTGAGGTTGACATCACAGGCA
	R: TGCTGTCAGAGGTCTTGTGCTC
Human *Aim2*	F: GCTGCACCAAAAGTCTCTCCTC
	R: CTGCTTGCCTTCTTGGGTCTCA
Human *Il6*	F: AGACAGCCACTCACCTCTTCAG
	R: TTCTGCCAGTGCCTCTTTGCTG
Human *Gapdh*	F: GTCTCCTCTGACTTCAACAGCG
	R: ACCACCCTGTTGCTGTAGCCAA
Human *Tnf Alpha*	F: CTCTTCTGCCTGCTGCACTTTG
	R: ATGGGCTACAGGCTTGTCACTC
Human *Il10*	F: TCTCCGAGATGCCTTCAGCAGA
	R: TCAGACAAGGCTTGGCAACCCA
Human *Il1b*	F: CCACAGACCTTCCAGGAGAATG
	R: GTGCAGTTCAGTGATCGTACAGG
Human *Il18*	F: GATAGCCAGCCTAGAGGTATGG
	R: CCTTGATGTTATCAGGAGGATTCA
Human *Casp4*	F: GGGATGAAGGAGCTACTTGAGG
	R: CCAAGAATGTGCTGTCAGAGGAC
Human *Il17a*	F: CGGACTGTGATGGTCAACCTGA
	R: GCACTTTGCCTCCCAGATCACA
Human *Ifng*	F: GAGTGTGGAGACCATCAAGGAAG
	R: TGCTTTGCGTTGGACATTCAAGTC
Human *Ifnb1*	F: CTTGGATTCCTACAAAGAAGCAGC
	R: TCCTCCTTCTGGAACTGCTGCA
Human *Ifna1*	F: AGAAGGCTCCAGCCATCTCTGT
	R: TGCTGGTAGAGTTCGGTGCAGA
Human *Il4*	F: CCGTAACAGACATCTTTGCTGCC
	R: GAGTGTCCTTCTCATGGTGGCT
