# Supplementary material for: Pulmonary inflammation and viral replication define distinct clinical outcomes in fatal cases of COVID-19
Source: PLoS Pathog. 2024 Jun 5;20(6):e1012222. doi: 10.1371/journal.ppat.1012222 (PMC11182505; doi:10.1371/journal.ppat.1012222)
Supplement: Attachment — Submitted filename: Response to Reviewers.docx [file ppat.1012222.s007.docx]

Point-by-point response to the reviewer’s question.

We thank the reviewers and the editors for spending the time to evaluate our work and for the constructive criticism.

We performed a substantial number of experiments, including *in vivo* infections in mice to address all the reviewer's concerns. We are certain that the addition of this new data significantly straightened the manuscript and we thank the reviewers and editors for the suggestions.

All the concerns and suggestions by the reviewers are addressed bellow and in the revised manuscript.

**Part I - Summary**

**Reviewer #1**: *Keyla et al. undertake an important study on lung autopsies from 47 individuals who succumbed to COVID-19 to identify underlying pathophysiological mechanisms of COVID-19 lethality. The authors identified several biomarkers of inflammation, with a focus on the NLRP3 inflammasome, that were elevated in tissues from COVID-19 compared to controls (benign regions of lung tissue from patients who died from lung adenocarcinoma). By co-staining tissues for markers of cell type and NLRP3 inflammasome activation, the authors suggest that inflammasome activation primarily occurs in monocytes/macrophages and endothelial cells, consistent with prior reports from experimental infections that inflammasome activation can occur in these cell types. Moreover, hierarchical clustering revealed that tissues from COVID-19 patients fell into two distinct groups: one characterized by lower inflammatory signatures, disseminated intravascular coagulation but higher viral loads, and one characterized by higher incidence of fibrosis and inflammation but lower viral loads. Collectively, the data suggest that COVID-19 deaths can occur from distinct processes, providing insights into disease etiology and potentially patient management.

The authors should be commended for completing this important study. However, my enthusiasm is somewhat diminished by some limitations of the current work. The entire manuscript is focused on analyses on post-mortem samples. While this led to some interesting observations (e.g., the increased incidence of NLRP3 inflammasome activation in macrophages/monocytes and endothelial cells and the identification of patient clusters), no additional studies were performed to verify these findings in experimental models of SARS-CoV-2 infection. As such, the authors’ findings are largely observational and correlative. Moreover, several prior studies on autopsy samples from patients that died from COVID-19 have been published (e.g., PMID: 34648357, 33033248, 33158808). References to these and other reports are largely lacking, and a meta-analysis, or at minimum, a narrative comparison of these prior studies with the current manuscript, which would allow for the authors to establish whether or not their major conclusions are generalizable, were not conducted. Finally, there are a few occasions where conclusions should be softened or contextualized. I have also made a few recommendations for additional analyses of existing data that may further inform some of the authors’ conclusions.*

Author’s Response: We thank the reviewer for the positive comments and constructive suggestions. As suggested, we generated significant new data that support the hypothesis that NLRP3 is involved in the genesis of the inflammation (as opposed to the hypothesis that NLRP3 is just an additional inflammatory marker elevated during the severe COVID-19). We performed experiments using transgenic mice expressing hACE2 and deficient in NLRP3 (and littermate controls) and performed infections in vivo. Our data show that compared to littermate controls, the *Nlrp3^-/-^* mice develop lower inflammatory processes, less severe disease, and succumb less to lethal COVID-19. These data strongly increase the relevance of our study as they provide data that support our assertion that the NLRP3 inflammasome operates in the genesis of the inflammatory process (as opposed to the hypothesis that the inflammasome is only an additional marker of disease gravity). All the other comments from the reviewer were addressed as indicated below.

***Reviewer #2:*** *In this study, the authors examined lung autopsies from COVID-19 patients and found that inflammasome activation correlates with COVID-19 severity. They also observed two mRNA expression patterns in lethal cases.

However, this manuscript suffers several serious defects.

Multiple studies have demonstrated the activation of inflammasomes in COVID-19 patients and its association with disease severity and clinical outcome. (including a study from the authors’ own group)
Nature volume 606, pages576–584 (2022) (*[*https://doi.org/10.1038/s41586-022-04702-4*](https://doi.org/10.1038/s41586-022-04702-4)*)
Nature volume 584, pages463–469 (2020) (*[*https://doi.org/10.1038/s41586-020-2588-y*](https://doi.org/10.1038/s41586-020-2588-y)*)
J Exp Med (2021) 218 (3) (*[*https://doi.org/10.1084/jem.20201707*](https://doi.org/10.1084/jem.20201707)*)
Same phenomenon is described again in Figure 2 of this manuscript, which should be attached as supporting information.*

Author’s Response: As mentioned by the reviewer, our group and others (Akiko Iwasaki, Judy Lieberman, Richard Flavell, etc.) reported the activation of inflammasome in COVID-19 and the role of NLRP3 inflammasome in aggravation of the disease (all these articles are referenced in our manuscript). However, this is not an article reporting inflammasome activation and its effects on COVID-19. Our study reports the existence of two distinct patterns of disease in lethal cases of COVID-19. To reach this conclusion, we performed a massive task force to quantify inflammasome activation in the autopsy of 47 patients. Regarding to Figure 2, we believe this figure is important because it confirms that illness time negatively correlates with viral loads and illness time positively correlates with NLRP3 puncta and ASC puncta in this cohort of patients.

*Furthermore, the authors claimed that inflammasomes are active in endothelial cells, but a study published last year also analyzing lung autopsy samples from COVID-19 patients, shows that there was no inflammasomes activation in endothelial cells. (Nature volume 606, pages576–584 (2022)) Therefore, further evidence is required to prove the presence of active inflammasomes in endothelial cells. More importantly, instead of discussing, the authors should provide experimental results concerning the c*

Author’s Response: We agree with the reviewer that using only material from post-mortem human samples makes it difficult to demonstrate that inflammasomes are activated in endothelial cells. It is possible that inflammasomes are released by other cells during pyroptosis and attach to endothelial cells. Thus, we decided to remove the data suggesting inflammasome activation occurs in endothelial cells. Importantly, the removal of this data from Figure 1 does not affect the novelty and relevance of the manuscript. The most important conclusion of the manuscript is the existence of two distinct patient clusters in lethal COVID-19 and this is not affected by the removal of endothelial cell data.

*The title and the conclusion of this manuscript are both misleading. Besides mRNA of inflammasome- related genes, the mRNA of many critical cytokines, such as IFNa, IFNb, IFNg, etc, is low expressed in the Cluster 1 as well. But the authors did not provide any evidence showing that inflammasome activation is the driving force behind the expression of these cytokines. Thus, inflammasome activation cannot be used to define the two clusters, let alone clinical outcomes.*

Author’s Response: This is a very important point that we addressed experimentally. We generated a transgenic hACE2 mouse deficient in NLRP3 (and littermate controls) and performed infections in vivo. Our data show that compared to littermate controls, the *Nlrp3^-/-^* mice developed lower inflammatory processes, less severe disease, and were more resistant to lethal COVID-19 (Fig. 6). These data increase the relevance of our study as it provides experimental demonstration to support our assertion that the NLRP3 inflammasome operates in the genesis of the inflammatory process (as opposed to the hypothesis that the inflammasome is only an additional marker of disease severity). We are certain that the addition of this data significantly improves the manuscript. The manuscript title was adjusted as suggested.

***Part II – Major Issues: Key Experiments Required for Acceptance*** *Please use this section to detail the key new experiments or modifications of existing experiments that should be absolutely required to validate study conclusions.

Generally, there should be no more than 3 such required experiments or major modifications for a "Major Revision" recommendation. If more than 3 experiments are necessary to validate the study conclusions, then you are encouraged to recommend "Reject".*

***Reviewer #1: (No Response)***

***Reviewer #2: (No Response)***

**Part III – Minor Issues: Editorial and Data Presentation Modifications**
Please use this section for editorial suggestions as well as relatively minor modifications of existing data that would enhance clarity.

**Reviewer #1**: Minor comments:
*- As described above, prior studies that performed similar analyses on lung autopsy samples from COVID-19 patients should be cited and discussed. If possible, a meta-analysis of published data to determine if the conclusions from this study are generalizable would greatly enhance the significance. It is appreciated that some of the analyses (e.g., inflammasome-specific biomarkers) are probably not available for cross-comparison.*

Author’s Response: As mentioned by the reviewer, there are 4 studies reporting inflammasome activation in autopsy of fatal cases of COVID-19 ({Junqueira, 2022 #698} (n=5 patients), {Rodrigues, 2021 #117} (n=5 patients), {Toldo, 2021 #148} (n=4 patients) and {Xian, 2021 #119} (n=11 patients). All these articles are all referenced in our manuscript as suggested.

*- Many clinical terms and biomarkers are incompletely described, which limits the accessibility of the manuscript to broad readership. To that point, upon trying to look up clinical definitions, it is not clear to me that observed levels meet established clinical criteria for some claims (e.g., platelet counts as an indication of DIC). Information should be provided to describe absolute levels in reference to established clinical definitions.
- If such patient data exists, it would be highly beneficial to provide biomarker data for patients prior to COVID-19 onset to assess ‘baseline’ levels of individual patients.*

Author’s Response: Unfortunately, we don’t have samples and data from patients prior to COVID-19 onset. As suggested by the reviewer, we have provided a better description of the biomarkers and criteria used define DIC.

*- Figure 1: adding labels to define cell populations would be helpful*

Author’s Response: As discussed before, this figure has been removed.

*- Figure 1: to the best of my knowledge, NLRP3 is not expressed in lung epithelia, including pneumocytes. Could NLRP3 staining in PDPN+ and SFTPC+ cells be background?*

Author’s Response: As mentioned to reviewer 2, we decided to remove the data suggesting inflammasome activation in these cells. Importantly, the removal of this data from Figure 1 does not affect the novelty and relevance of the manuscript. The most important conclusion of the manuscript is the existence of two distinct patient clusters in lethal COVID-19 and this is not affected by the removal of endothelial cell data. Regarding the possibility that the inflammasomes shown are background, our data showing the colocalization of NLRP3 and ASC in the puncta (Fig. 1 H, I) provides evidence that the antibodies are specifically detecting inflammasome puncta.

*- Line 148 (and elsewhere): when referring to inflammasome activation, the authors should be specific when they intend to refer to the NLRP3 inflammasome. Not all inflammasomes utilize ASC, and not all cell types express NLRP3. Moreover, other inflammasomes have been implicated in the response to SARS-CoV-2.*

Author’s Response: We have adjusted that.

*- The antibody used to stain for IL-1beta detects both the pro- and active p17 form. Thus, that data is largely uninformative.*

Author’s Response: We agree with the reviewer that in the immunohistochemistry experiment shown in Supplementary Fig. 3, we can’t infer inflammasome activation using IL-1β. In this particular experiment, we used anti-IL-1β to demonstrate that these cells express pro-IL-1β (and all other inflammasome components). Inflammasome activation is assessed by scoring puncta formation, which is measured in many figures in the manuscript.

*- Line 176: the correlation between NLRP3/ASC staining and time of disease (Fig 2C and 2D) is pretty weak. The conclusion should be measured or contextualized.*

Author’s Response: We thank the reviewer for the comment and we agree that the correlation between NLRP3/ASC staining and time of disease is weak, but we believe it is important to show the data. We believe the correlation is weak because of the existence of the two different clusters, a point that follows up in the next figure.

*- The conclusion from Supplementary Fig 5 is unclear. Some correlations exist, but transcriptional correlations do not capture the state of inflammasome signaling.*

Author’s Response: We agree with the reviewer and we clarified this in the text. This figure (now Supplementary Fig. 3) indicates gene expression (not inflammasome activation) and we believe it is important to show this data as a supplementary figure.

*- Fig 3B and 3C: it is unclear to me why the data is stratified by different x-axis variables*

Author’s Response: These two figures show different x-axis because Aa gradients are only measured in patients in ICU. Thus Fig. 3C only shows patients under mechanical ventilation whereas Fig. 3B includes data from all patients and therefore we used illness time in the x-axis.

*- Line 242: the word ‘dictate’ in the heading is inappropriate*

Author’s Response: We have adjusted that

*- Given that cluster 2 is characterized by vascular dysfunction, it seems reasonable to reanalyze the data from Fig 1I-P across samples from cluster 1 and cluster 2 to determine if the incidence of inflammasome activation in endothelial cells is higher in cluster 1.*

Author’s Response: We performed these analyses as suggested by the reviewer but we did not detect statistically significant differences, possibly because splitting the patients reduces the number of samples and affects the statistical power.

*- Although the mechanism ventilation data is negative data, it should still be presented in the Results, and then discussed in the Discussion.*

Author’s Response: We have done that.

*- Line 337: the authors should be careful to not imply that cells with markers of SARS-CoV-2 were infected by the virus – as the authors discuss elsewhere, it’s possible, particularly for phagocytic cells, that spike and other indicators are due to virus uptake or engulfment as opposed to infection.*

Author’s Response: We agree with the reviewer and have adjusted that.

*- Line 352: should also cite PMID: 36129453.*

Author’s Response: As discussed previously, we removed the Fig. 1 and endothelial cell data. Thus the discussion about inflammasome activation in endothelial cells were also deleted from the manuscript.

*- A better description of the criteria for scoring CXR in the Methods would be helpful.*

Author’s Response: We have provided a more detailed description of the scoring CXR in the Methods.

***Reviewer #2: (No Response)***
