# Supplementary material for: Pulmonary inflammation and viral replication define distinct clinical outcomes in fatal cases of COVID-19
Source: PLoS Pathog. 2024 Jun 5;20(6):e1012222. doi: 10.1371/journal.ppat.1012222 (PMC11182505; doi:10.1371/journal.ppat.1012222)
Supplement: Attachment — Submitted filename: Response to Reviewers.docx [file ppat.1012222.s008.docx]

Point-by-point response to the reviewer’s question.

**Part I - Summary**

**Reviewer #1**: *The authors satisfactorily responded to and amended the issues I had with their description of methodology and they appropriately contextualized claims and softened conclusions. I also agree with the authors that removing aspects of Figure 1 does not lower the impact or novelty.

The authors have now also included an analysis comparing SARS-CoV-2 infection of hACE2+ WT and Nlrp3-/- mice. As has been reported elsewhere, the authors found that Nlrp3-/- mice exhibit a reduced inflammatory disease compared to WT mice, based on observed differences in parenchyma area, survival, and overall clinical score. Other indicators, such as inflammatory cytokines and weight loss *trend* in a manner consistent with Nlrp3 inflammasome involvement in disease, but in the present study are not overwhelmingly convincing. It appears the data may represent a single experiment, although it is not clear from the legend or methods.*

*The original finding of interest is that individuals that died due to COVID-19 cluster into two groups, suggesting distinct etiology. One of these clusters had hallmarks of inflammasome-mediated pathogenesis. However, since these are post-mortem analyses, the role of Nlrp3 in the development and progression of disease is only speculative. So while the new data does provide a more direct assessment of the role of Nlrp3 in SARS-CoV-2-associated inflammatory pathogenesis in mice, it feels like a missed opportunity to further substantiate the post-mortem findings. For example, one might predict that the Nlrp3-/- mice (that do not survive) may have profiles similar to cluster 1, but analyses to compare readouts that stratified human cluster 1 and 2 in the mice other than fibrosis by H&E (e.g., gene expression, viral load, etc) were not performed. To that end, I strongly encourage the authors to be very clear about what can and cannot be gleaned from the mouse study.*

Author’s Response: Based on the reviewer's suggestions, we conducted additional experiments with hACE2 transgenic mice that were sufficient and deficient for NLRP3. We now present robust data employing a substantial number of mice (n=14 *Nlrp3*^+/+^ and n=15 *Nlrp3*-/-), enabling us to demonstrate that NLRP3 contributes to promoting disease exacerbation in an experimental model of SARS-CoV-2 infection. Regarding the evaluation of fibrosis in infected mice, this mouse model of infection does not induce fibrosis as observed in patients, possibly due to the duration of infection. To achieve this goal, another infection model should be developed, perhaps one with a longer duration, but believe this is beyond the scope of this manuscript. Thus, we followed the reviewer's suggestion and acknowledged the limitations of our in vivo infection model in the manuscript. We thank the reviewer for their thoughtful evaluation of our manuscript in the first and second rounds of revision.

***Reviewer #2:*** *The revised manuscript is primarily focused on characterizing two distinct patterns observed in lethal cases of COVID-19. By utilizing hACE2 transgenic mice, the authors further provided experimental evidence showing the involvement of NLRP3 inflammasome in COVID-19 aggravation, which could potentially recapitulate the observation in the two patient clusters in mouse model of COVID-19. While this revised version is of interest, I believe this manuscript could be considerably strengthened by addressing the comments below.

In all mice studies, each group should include a minimum of five mice, which results in more compelling data, particularly when dealing with significant variations/error, as seen in Fig6 H/I.*

*To better reflect the varying activation levels of NLRP3 in the two clusters of patients, mice of Nrp3+/- heterozygous should be included in the experiments of Fig6 B/C/D/E/F. In addition, Fig6 G/H/I should add the Nlrp3+/+ group.*

Author’s Response: Based on the reviewer’s suggestions, we conducted additional experiments with hACE2 transgenic mice that were sufficient and deficient for NLRP3. We now present robust data, employing a substantial number of mice, enabling us to demonstrate that NLRP3 accounts for disease exacerbation in an experimental model of SARS-CoV-2 infection. Regarding the suggestion to use +/- mice, we decided to remove this experimental group to avoid misleading intermediate phenotypes and because the addition of +/- mice would lead to a reduction of +/+ and/or -/- mice due to space limitations in our animal BSL3 facility. Instead, we increased the number of +/+ and -/- mice. Our new figures 6D-E now show a total of 14 *Nlrp3*^+/+^ and 15 *Nlrp3*^-/-^ mice, providing us with a solid dataset to perform statistical analysis and reach significant diferences.

*It would not be unexpected that a mouse model cannot fully mimic patient pathophysiology. Therefore, results of other cytokines, such as IFNa/b, IL-1, IL-18 etc., should also be included in Fig6 to better define the responses in mice with different Nlrp3 expression levels.*

Author’s Response: As previously mentioned, we excluded the +/- data and the cytokine in mice. The rationale behind incorporating a mouse model of SARS-CoV-2 infection (included during the first round of revisions following reviewers' suggestions) was to examine the cause-and-effect relationship of NLRP3 on disease development. This mouse data complements the correlational observations inherent in data obtained from human samples. It is important to mention that our data obtained from human samples does include cytokine quantifications, so additional experiments to quantify cytokines in mice would not help to understand the observations in humans. Therefore, we are confident that the removal of +/- animals and cytokine data does not impact the relevance and novelty of the manuscript. By contrast, the addition of robust experimental groups composed of *Nlrp3*^+/+^ and *Nlrp3*^-/-^ mice strengthens the mouse data and supports our conclusions obtained from human samples.

*Minor points
The caption of fig6 is a bit confusing, especially in the description of mice’s genetic background.*

Author’s Response: We have addressed this, and we thank the reviewer for pointing this out.

*Line 112, use “mechanical ventilation” instead of the abbreviation “MV” when it first appears in the text.*

Author’s Response: We have done this.

***Part II – Major Issues: Key Experiments Required for Acceptance*** *Please use this section to detail the key new experiments or modifications of existing experiments that should be absolutely required to validate study conclusions.

Generally, there should be no more than 3 such required experiments or major modifications for a "Major Revision" recommendation. If more than 3 experiments are necessary to validate the study conclusions, then you are encouraged to recommend "Reject".*

***Reviewer #1: (No Response)***

***Reviewer #2: (No Response)***

**Part III – Minor Issues: Editorial and Data Presentation Modifications**
Please use this section for editorial suggestions as well as relatively minor modifications of existing data that would enhance clarity.

**Reviewer #1**: Minor comments:
*- Cytokine analysis for mouse studies is missing in the methods section.

- The concluding sentence of the results (line 289-292) should include the term “…affects the disease progression [in mice]…” and “…observed in [some] lethal cases of COVID-19.” i.e., it’s certainly not all cases, given the authors conclusions regarding cluster 1 and cluster 2.*

Author’s Response: Thank you for pointing this out. These points have been adjusted.

***Reviewer #2: (No Response)***
